# Effects of Clicker Training on Behavioral and Stress Markers of Welfare in the F1 Generation of CD1 Mice: A Pilot Study

**DOI:** 10.3390/ani16111642

**Published:** 2026-05-27

**Authors:** Sandra Reichel, Fernando Gonzalez-Uarquin, Dorothea Pichl, Konstantin Radyushkin, Jan Baumgart, Nadine Baumgart

**Affiliations:** TARCforce3R Center, University Medical Center of the Johannes Gutenberg University, 55122 Mainz, Germany

**Keywords:** clicker training, laboratory mice, PRT, animal welfare, behavioral tests, corticosterone, 3R, refinement techniques

## Abstract

Training in laboratory animals is important to ensure that scientific studies are reliable, reproducible, and ethically acceptable. Well-trained animals experience less stress and exhibit fewer unwanted behaviors, improving both welfare and research outcomes. Clicker training is widely used in animal training as a positive reinforcement method to reduce distress. The present study was designed as a pilot study to examine the effects of a four-day clicker training protocol applied to both dams and their offspring, with behavioral outcomes assessed exclusively in the offspring. The results indicated that clicker training potentially increased voluntary interaction with the experimenter and promoted body weight gain during the training period. No significant effects of offspring training were found for classical anxiety-related measures (EPM open arm time, OF center time, Nest Building Test, Sucrose Preference Test, Forced Swim Test) or plasma corticosterone. A potential sex effect was observed across locomotor, center-zone, and corticosterone measures. Maternal training effects on offspring outcomes should be interpreted as preliminary exploratory observations, as only two dams per maternal group were available. Given the exploratory nature and limited sample size of this pilot study, the findings should be interpreted with caution. Under the present condition, clicker training produced a context-specific improvement in human–animal interaction without evidence of harm, but did not produce generalized reductions in anxiety-like behaviors as assessed by standard paradigms. Further studies with larger sample sizes are needed to confirm these findings.

## Simple Summary

Training methods have become increasingly important for laboratory animal welfare, with clicker training representing a specific and widely used form of positive reinforcement training (PRT). Clicker training has been shown to improve animals’ quality of life by increasing voluntary interactions with experimenters, which are associated with reductions in anxiety-related behaviors. This pilot study investigated the potential impact of clicker training as a form of PRT on behavioral and physiological stress markers in F1-generation Crl:CD1(ICR) mice without a stressor. The findings indicate that clicker training could enhance human–animal interactions in F1-generation without decreasing animal welfare, supporting clicker training potential to refine laboratory animal research and improve welfare outcomes.

## 1. Introduction

Animal models play a fundamental role in advancing biomedical research by enabling the investigation of complex biological processes and the development of new therapies [1]. Within the implementation of the 3Rs (Refinement, Reduction, and Replacement), refinement strategies are aimed at minimizing pain, distress, and discomfort to enhance animal welfare [2]. Despite ongoing efforts to refine experimental procedures, laboratory animals often exhibit stress- and anxiety-associated behaviors during handling (e.g., picking up mice by tail), which can negatively impact both animal welfare and data quality by introducing variability, reducing reproducibility, and prolonging experimental tasks [3].

Methods that reduce stress while maintaining scientific rigor are essential for improving both animal welfare and the quality of scientific data [4]. Positive reinforcement training (PRT), and specifically clicker training, has been shown to reduce stress and improve cooperation with handlers in various animal species [5,6,7]. Positive reinforcement training is a form of operant conditioning that enables the investigator to systematically shape animal behavior. In this approach, the performance of a desired behavior is immediately followed by a positive stimulus, typically a food reward, with the aim that the animal forms an association between the behavior and its rewarding outcome. Over time, this reinforcement increases the likelihood that the behavior will be repeated. Clicker training represents a specific application of positive reinforcement training that incorporates a conditioned secondary reinforcer—the characteristic “click” sound. In this context, the clicker functions as a secondary reinforcer, while the food reward serves as the primary reinforcer. Through repeated pairings, the animal learns that the clicking sound reliably predicts the delivery of a reward [8]. More specifically, the click acts as a precise “time bridge” between the behavior and the subsequent reward [9]. The trainer produces the click at the exact moment the desired behavior occurs, thereby marking the behavior without delay [10], and then follows it with the food reward. This temporal precision is critical, as it allows the animal to clearly associate the reward with the specific action being performed. As a result, the marked behavior is reinforced and increases in frequency over time [8,9,11]. In this pilot study, mice were specifically trained to voluntarily enter a handling tunnel, with the aim of reducing handling-induced stress by allowing the animals to initiate contact with the experimenter voluntarily.

In rodents, clicker training can reduce anxiety-related behaviors, support voluntary participation, and may contribute to improved animal welfare in laboratory settings [11,12,13]. Most existing research on clicker training has focused on its immediate effects on the animals directly trained. However, there is a significant gap in knowledge regarding whether clicker training benefits extend to the following generations. Specifically, it is not yet clear if training mothers can produce lasting welfare improvements in their offspring.

In mice, natural variations in maternal care have been shown to induce long-lasting changes in stress reactivity, emotional behavior, and hypothalamic–pituitary–adrenal (HPA) axis regulation in the offspring [14]. Cross-fostering studies in mouse strains further demonstrate that early postnatal maternal environment significantly shapes anxiety-like behavior and basal corticosterone levels later in life [15].

In addition, mice are capable of social learning, suggesting that task-relevant behaviors may be transmitted from dam to offspring through observational mechanisms. Consistent with this, the findings reported in the study [15] indicate that certain behavioral advantages can be transmitted across generations without requiring direct genetic inheritance. Instead, the data suggest that these effects are primarily mediated by parental behavior, social interactions, and environmental conditions. In particular, the quality of care and early-life experiences of the offspring play a crucial role in determining whether observed advantages persist into the next generation. This form of transmission should therefore be understood as an intergenerational transfer of experience rather than a stable biological inheritance. In other words, offspring may benefit from conditions shaped by their parents, but these effects are not necessarily embedded in a lasting or self-sustaining biological mechanism across generations.

Concerning learning processes, studies in mice demonstrate that behavior can indeed be shaped by experience and, to some extent, influence subsequent generations. However, this influence occurs predominantly through indirect pathways, such as altered parental care or environmental structuring, rather than through the inheritance of learned information itself. Thus, learning in the strict sense is not directly heritable, but instead gives rise to context-dependent effects that depend strongly on the surrounding conditions.

Importantly, these mechanisms cannot be readily generalized across all species. While mice as model organisms are particularly sensitive to environmental and social influences, the extent and stability of such intergenerational effects may vary substantially between species [16]. Overall, current evidence suggests that the transmission of advantages across generations is best explained by experience- and environment-dependent processes, rather than by a universal mechanism of inheriting learned behaviors [16].

The existing gap in the literature is whether positive reinforcement can be transferred to the F1 mouse generation by training the dams. Given that, this exploratory pilot study addresses the mentioned gap by investigating the generational effects of clicker training in laboratory mice. The research aims to systematically measure potential behavioral and physiological stress indicators in the F1 generation of Crl:CD1(ICR) mice born to trained and untrained mothers. We specifically explore whether maternal training may influence stress responses in offspring, which could reduce the need to individually train each animal, making the technique more practical and attractive for researchers. The offspring of trained mothers might benefit from enhanced maternal care and social learning, potentially requiring only brief reinforcement training themselves.

The objectives of this pilot study were fourfold: (1) To establish and implement a standardized clicker training protocol for Crl:CD1(ICR) mice; (2) To evaluate the effects of this training on behavioral indicators of stress and anxiety; (3) To assess physiological stress markers—plasma corticosterone concentrations—in trained and untrained animals; (4) To explore whether potential effects of clicker training in the parental generation may also be reflected in their offspring (F1), particularly in terms of possible reduced stress reactivity and potentially improved task participation following minimal additional training.

We hypothesized that clicker training may lead to a reduction in behavioral and physiological stress indicators in trained Crl:CD1(ICR) mice compared to untrained controls, consistent with previous findings showing that positive reinforcement training can reduce stress and improve handling outcomes in laboratory animals [17]. Furthermore, we explored whether maternal clicker training might indirectly influence stress responsiveness and task engagement in the F1 generation. Maternal environment and stress exposure are known to affect offspring behavior and stress reactivity through both behavioral and physiological pathways [18,19]. Therefore, offspring of trained mothers may exhibit lower stress reactivity and improved task performance, potentially requiring less reinforcement training. However, as evidence for transgenerational effects of positive reinforcement training in laboratory mice remains limited, this aspect of the study should be considered exploratory. If such effects were present, they could suggest an additional welfare benefit and improve the practicality of refinement strategies in laboratory animal research.

## 2. Materials and Methods

This study was conducted in accordance with the ARRIVE 2.0 guidelines [20]. All experimental protocols and animal management procedures received prior approval from the Rhineland-Palatinate State Authority (permit number: G-20-1-131), in compliance with the European Directive 2010/63/EU on the protection of animals used for scientific purposes.

### 2.1. Mice

Seven Crl:CD1(ICR) mice (four 7-week-old female mice and three 7-week-old male mice) were acquired from a verified breeder (Charles River, Sulzfeld, Germany). Following acclimatization, two female mice were trained according to the protocol described in 2.2. while two females remained untrained as controls, trained. All four primiparous females were subsequently paired with male conspecifics for breeding. At seven weeks of age, each litter (*n* = 47; 21 male, 26 female) were randomly assigned to one of four experimental cohorts: M+O+ (trained mothers with trained offspring, *n* = 12), M−O+ (untrained mothers with trained offspring, *n* = 12), M+O− (trained mothers with untrained offspring, *n* = 10), and M−O− (untrained mothers with untrained offspring, *n* = 13). The choice of a four-group design (M−O−, M+O+, M−O+, M+O−) for studying offspring allows for a differentiated analysis of the effects of parental and offspring training. The M−O− group serves as an untreated control group and enables the assessment of baseline levels in terms of behavior and physiological parameters (e.g., stress/cortisol). It therefore indicates whether the training interventions overall have an effect compared to the baseline condition. In the M−O+ group, only the offspring are trained. This group serves as a direct comparison with the other groups to determine whether individual training of the offspring or parental influences are more effective in producing the observed behavioral and physiological changes. The M+O− group, in which only the mothers are trained, allows the investigation of intergenerational effects without a direct training influence on the offspring. In addition, this design can lead to a considerable saving of time and resources, as only the number of mothers needs to be trained. If this condition were to show effects comparable to offspring training, a reduction in maternal training alone could be considered for future studies. The M+O+ group combines both training conditions (mothers and offspring). It is used to examine whether a cumulative effect of training exists, i.e., whether double exposure leads to stronger changes in behavior and cortisol levels than single training conditions.

Maternal treatment (trained vs. untrained) was used as a factor to ensure a balanced distribution within maternal treatment groups and to enable the assessment of transgenerational effects. As sex was unknown before birth, group allocation was adjusted after birth to achieve an approximately equal distribution of males and females across experimental groups. 24 mice from the progeny were trained at seven weeks of age, while 23 were kept as controls for further comparisons. All mice were maintained in accordance with the recommendations of the Federation of Laboratory Animal Science Associations [21]. Animals were housed in same-sex groups of two or three in type II long filter-top cages (Tecniplast, Buguggiate, Italy; SealSafe Plus, polyphenylsulfone, 36.5 cm × 21 cm × 14 cm), each equipped with two paper tissues for nesting, a red transparent shelter (Tecniplast, Buguggiate, Italy; 5.5 cm × 14 cm), and opaque 10 cm × 3.5 cm PVC tunnels (HT pipe DN 5, hardware store) which also served for animal-friendly handling and transfer of the animals. The animal facility was maintained on a 12 h light/dark cycle (200 lux from 07:00 to 19:00) in a temperature- and humidity-controlled environment (22–24 °C and 50–55%, respectively). Autoclaved water and standard chow (Maus-Zucht pellets V1124–300, ssniff, Soest, Germany) were provided ad libitum.

### 2.2. Clicker Training Protocol

Mothers and offspring underwent identical clicker training protocols over four consecutive training days, with two days of food presentation, behavioral and physiological assessments conducted exclusively on the F1 generation to evaluate transgenerational effects of maternal training on stress-related outcomes.

Before training initiation, subjects were habituated to the food reward (Brinkers Chocolate Symphonie no. 3 white chocolate cream, Enschede, The Netherlands) by placing it in home cages overnight for two consecutive days. For administration, a double spatula (ROTILABO^®^ micro, 150 mm, 4 mm, Carl ROTH, Karlsruhe, Germany) was used to smear the chocolate onto at least two cage walls; the applied amount corresponded to one spatula-width per application [22]. The criterion for confirming reward intake consisted of visual verification of complete consumption on both mornings before the start of training.

After the two days of reward introduction, the clicker training protocol was conducted over four days (Table 1). The protocol was based on previous reports [11,23] with some modifications. We aimed to shorten the original [11,23] protocol to improve its practical feasibility and integration into experimental workflows with reduced duration, as the original three-week training protocol is not readily applicable in routine practice or scalable to larger cohorts of animals. Briefly, the clicker introduction session was performed individually for each mouse over five minutes to establish a conditioned association between the auditory “click” stimulus and food reward delivery: (1) During preparation, the trained mouse remained in its home cage while cage mates were temporarily relocated to a separate holding cage equipped with familiar enrichment materials to prevent environmental interference. (2) Each session started with a 30 s acclimatization period without training material or enrichment (Tunnel) present. After the 30 s acclimatization period, the PVC tunnel was positioned along the cage wall to exploit natural thigmotactic behavior. (3) The mice were allowed to voluntarily enter the tunnel without coercion, and upon tunnel entry, an immediate click sound was generated, followed by food reward presentation. Once feeding behavior was initiated, ten consecutive clicks were delivered before reward removal, with multiple tunnel-entry opportunities provided within each session to establish reliable stimulus-response associations.

Following the introduction session, daily training sessions lasted 3.5 min per subject, structured as follows: (1) Pre-session acclimatization consisted of a 30 s habituation period without training equipment present. (2) Three training intervals alternated between 45 s active training periods during which the tunnel was available for voluntary entry and 15 s inter-trial intervals with complete equipment removal.

Food rewards were presented for single-bite consumption only to maintain motivation across trials, with immediate removal following consumption to prevent satiation effects. One training session was conducted per day for each mouse, with consistent timing maintained to preserve circadian rhythm considerations. This operant conditioning paradigm established reliable conditioned associations through temporal contiguity while minimizing handling stress and maintaining ecological validity through voluntary behavioral expression.

The behavioral analysis was conducted exclusively on the offspring, as the primary aim of the study was to investigate the transferability of clicker training across generations. The maternal training status (trained vs. untrained) served solely to establish the experimental groups and was not itself analyzed in detail.

### 2.3. Voluntary Interaction Test

The voluntary interaction test quantifies voluntary human–animal interaction using a validated protocol adapted from Hurst and West [24]. Testing was conducted on Day 0 (pre-training) and Day 7 (post-training) with mice maintained in their established social groups.

**Figure 1:**
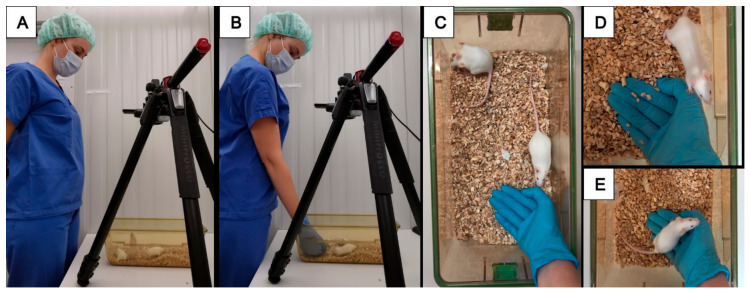
Experimenter conducting the interaction test (**A**,**B**) and scoring the mouse behavior (Sniffing: (**C**), Touching: (**D**), and Climbing: (**E**)).

**Figure 2:**
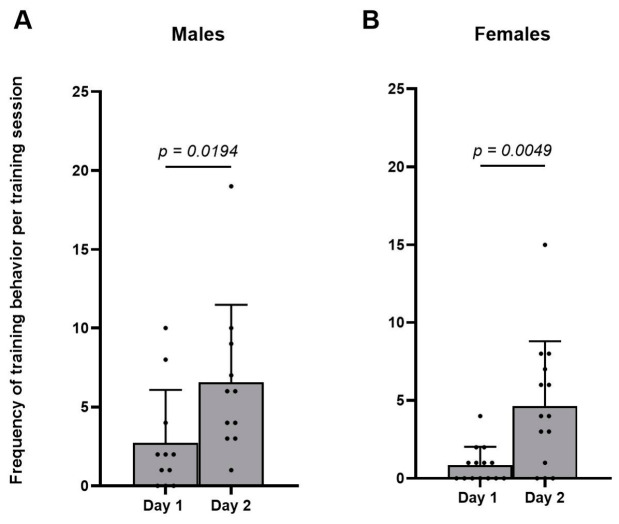
Frequency of training behavior in 7-week-old Crl:CD1(ICR) male (**A**) and female (**B**) offspring. It shows the first two days of training. We used a nonparametric test (Mann–Whitney U test) for statistical analysis, *n* = 11/*n* = 14. Each point represents an individual. Bars indicate the means ± SD. The exact *p*-values are provided when significant differences are given.

Cage enrichment items (lid, grid, tunnel, shelter, nesting material) were removed, and animals were color-marked on the tail for video identification. Using a Sony 4K camera (SONFDRAX53BL, Tokyo, Japan) under standard lighting, two consecutive 60 s phases were recorded: (1) baseline observation with the experimenter standing motionless in front of the cage (Figure 1A), and (2) hand-presentation phase with the experimenter’s gloved hand placed stationary on the bedding in the front cage area (Figure 1B).

**Figure 3:**
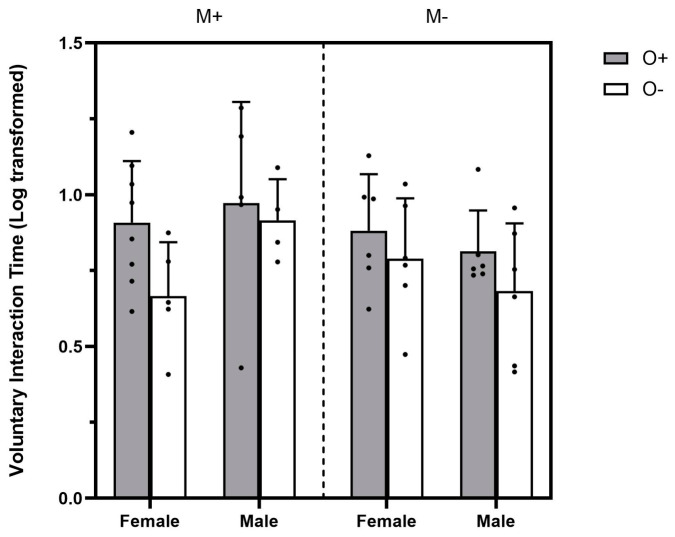
Determination of the voluntary interaction time between male and female offspring with the experimenter. We transformed the data and used three-way ANOVA for statistical analysis and multiple comparisons. The training of the offspring had an influence on the voluntary interaction time. The interaction between sex and the training of the mothers’ influences the voluntary interaction time as well. M-O- group (*n* = 12), M+O- group (*n* = 10), M-O+ group (*n* = 12), and M+O+ group (*n* = 13). Each point represents an individual. Bars indicate the mean ± SD.

Videos were analyzed using XNote Stopwatch software (dnSoft Research Group, v1.60, 2018) to measure. We scored the following parameters in the voluntary interaction test: (1) Sniffing (Figure 1C), (2) Touching (Figure 1D), and (3) climbing onto the experimenter’s hand (Figure 1E). Total interaction time represents the cumulative duration across all three behaviors during the 60 s hand-presentation phase.

These parameters were consolidated into total interaction time for statistical evaluation of training regarding the interaction between mice and the experimenter.

### 2.4. Weight Gain

Body weight was measured using a precision analytical balance (Sartorius ED822-CW, Göttingen, Germany) immediately following the voluntary interaction test. Individual mice were transferred to a weighing bowl using the tunnel handling method to minimize stress-induced weight fluctuations. The weighing bowl was sanitized with 70% ethanol between each cage group and allowed to air-dry completely to prevent chemical contamination and ensure accurate measurements.

### 2.5. Elevated Plus Maze (EPM) Test

We used a self-constructed Elevated Plus Maze (65 cm × 65 cm × 62 cm, L × W × H) under standardized conditions at 09:00 AM. Mice were transported from their housing room to the testing room and allowed to acclimatize for 30 min. During this period, the maze was centered beneath a bi-color ring light (Walimex Meadow 960 Pro BiColor, 220 V, Wiernsheim, Germany) adjusted to 220 lux (Voltcraft LX-1108 lux meter, Bavaria, Germany), and a Sony CX240E Handycam (Tokyo, Japan) was mounted vertically and centrally above the apparatus to record without casting shadows. Before each mouse, the maze was thoroughly cleaned with 70% ethanol and air-dried.

Each trial comprised the following sequence: (1) the mouse was released into one open arm via a PVC tunnel positioned so its exit lay no more than 3 cm above the arm, ensuring a single exit choice; (2) upon voluntary emergence, recording started and continued for 5 min while the experimenter remained motionless and out of sight; and (3) at the end of recording, the tunnel was reinserted, and the mouse was allowed to voluntarily re-enter before being returned to its home cage via the tunnel. Video files were subsequently analyzed using EthoVision XT (Noldus Information Technology, Wageningen, The Netherlands) to quantify the duration spent in open versus closed arms and derive anxiety-related indices.

### 2.6. Open Field (OF) Test

We used a self-constructed open field arena (40 cm × 40 cm × 40 cm, L × W × H) under standardized conditions at 09:00 AM. Mice were transferred from their housing room to the testing room and allowed to acclimatize for 30 min while the test was prepared. During this interval, a Sony CX240E Handycam^®^ (Tokyo, Japan) was mounted vertically and centrally above the arena on a Manfrotto 432-2,7 autopole (Bassano del Grappa, Italy), and illumination was adjusted to 220 lux using a lux meter (sensor facing up in the arena) to eliminate shadows. The arena was cleaned with 70% ethanol and air-dried before each mouse.

Each trial started when the mouse was placed in a corner of the arena via a PVC tunnel; as soon as the animal voluntarily went out, the tunnel was removed, and a 5 min recording began. Throughout the session, the experimenter remained motionless and out of the animal’s line of sight. At the end of 5 min, recording ceased, and the mouse was guided back into the tunnel to return to its home cage. Behavioral tracking and quantification of total distance traveled, time spent in the center versus periphery, and locomotor parameters were performed offline using EthoVision XT Noldus Information Technology, Wageningen, The Netherlands.

### 2.7. Nest Building (NB) Test

Nest building was assessed individually in freshly prepared Type II cages (ZOONLAB GmbH, Castrop-Rauxel, Germany; 26 cm × 20 cm × 14 cm, L × W × H) using pressed cotton “Nestlets” (Datasand No. 14010, Castrop-Rauxel, Germany) weighing 2.48–2.52 g. Cages were prepared either on the test day or the day before by removing all enrichment items and placing a single Nestlet in each cage. Mice were introduced at the onset of the dark phase and left undisturbed for 12 h, after which nests were scored by Deacon [25] by observers blinded to treatment groups, and the nests were photographed. Nest building in mice is assessed using a 1–5 scoring system based on how much of the Nestlet (cotton square) is torn and how the material is arranged:

1. Score 1: Nestlet mostly untouched (>90% intact).
2. Score 2: Partially torn (50–90% intact).
3. Score 3: Mostly shredded (<50% intact), but cotton is spread around rather than gathered into a nest.
4. Score 4: Identifiable nest: >90% shredded and material gathered into a nest within a quarter of the cage, but walls are low (covering <50% of nest circumference).
5. Score 5: Near-perfect nest: >90% shredded, formed into a crater with walls taller than a mouse’s body for >50% of circumference.

Because nest building is variable, sometimes intermediate scores (e.g., 4.5) are assigned if a nest is nearly perfect, but some Nestlet remains unshredded.

### 2.8. Sucrose Preference (SP) Test

All measurements were performed under controlled laboratory conditions (22 ± 2 °C, 55 ± 10% relative humidity, 12 h light/12 h dark cycle), and bottles were refilled with freshly prepared solutions before each habituation and test phase.

Adult rodents were housed individually in Type II cages and acclimated to the testing environment prior to experimentation. A two-bottle choice paradigm was employed over a three-day period [22]. Two transparent glass bottles were affixed to each cage via partitioned grid panels; one bottle contained tap water and the other a 1% (*w*/*v*) sucrose solution (D(+)-Saccharose; Carl Roth GmbH, Cat. No. 9097, Carl ROTH, Karlsruhe, Germany. The sucrose bottle was identified by a green dot label to prevent misidentification.

On Days 1 and 2, animals underwent habituation sessions to familiarize them with the dual-bottle configuration and the sucrose solution. Each day at 6:00 PM, bottles were presented in one orientation (sucrose on the right, water on the left). After 12 h, at 6:00 AM, bottle positions were swapped (sucrose on the left, water on the right). This counterbalancing was performed to mitigate side bias.

On Day 3 at 6:00 PM, the bottles were arranged in the original setting (sucrose right, water left), and their initial masses were recorded to the nearest 0.01 g. Following a 12 h measurement phase concluding at 06:00 AM, final bottle masses were obtained. Sucrose intake (g) was calculated as the difference between the starting and final mass of the sucrose solution bottle; water intake was determined similarly. Total fluid intake was defined as the sum of sucrose and water intake. Sucrose preference (%) was calculated using the formula:

*Sucrose Preference* = (*Sucrose Intake*/*Total Fluid Intake*) × 100

### 2.9. Forced Swim (FS) Test

The forced swim apparatus consisted of a 1500 mL glass beaker (VWR, catalog no. 213-0470, Darmstadt, Germany) filled with tap water maintained at 23–25 °C. Water depth was set to prevent mice from touching the bottom. The water was completely replaced after every third mouse to ensure consistency in temperature and hygiene. A high-definition 4K video camera (Sony SONFDRAX53BL, Tokyo, Japan) was mounted on a tripod at the level of the beaker’s midpoint.

Testing started at 9:00 AM. Mice were transferred singly from the housing room to the experimental room immediately before testing. Each mouse was gently grasped by the base of the tail and lowered headfirst into the water; the timer was started at the moment the tail left the experimenter’s hand.

The swimming session lasted 6 min [26]. Throughout the trial, the experimenter remained seated quietly behind an opaque partition to intervene only if necessary. If a mouse exhibited continuous immobility—defined as floating without active limb or tail movement—for more than 30 s, the session was terminated, and the mouse was immediately removed, as required by the responsible regulatory authority for animal welfare reasons. Otherwise, mice were allowed to swim for the full 6 min. We excluded 9 mice from the FST (male: M−O− *n* = 2, M−O+ *n* = 1, M+O+ *n* = 1; female: M−O+ *n* = 3, M+O− *n* = 1, M+O+ *n* = 1) because of the protocol, which stopped the test after 30 s of immobility. All staff involved in collecting data in the main study protocol were blinded whenever possible.

Upon completion or early termination of the trial, recording was stopped, and each mouse was retrieved using the hand-cupping method. Excess water was gently dabbed from the fur with lint-free paper towels before the mouse returned to its home cage. All equipment was sanitized with 70% ethanol and wiped dry between subjects. Swims were scored offline for latency to immobility and total immobility time using behavioral analysis software (EthoVison XT, Version 15, Noldus Information Technology, Wageningen, The Netherlands).

### 2.10. Plasma Corticosterone

Blood samples were collected approximately 15 min after completion of the FST by euthanasia using compressed carbon dioxide (CO_2_) inhalation (Constant chamber filling speed/influence rate of 100% CO_2_ is at least 30%–max. 70% of the chamber volume/minute). All corticosterone values, therefore, reflect acute post-swim stress reactivity and must not be interpreted as indicators of resting HPA-axis function or chronic welfare status. Blood samples were collected immediately via cardiac puncture, with a minimum volume of 200 μL obtained from each animal and transferred to EDTA K3E anticoagulant tubes (Sarstedt, catalog no. 41.1504.005, 1.3 mL capacity, Sarstedt, Germany). Samples were placed on ice immediately following collection to preserve sample integrity.

Blood samples were centrifuged at 3000× *g* for 10 min at 4 °C to separate plasma from cellular components. The resulting plasma was carefully aspirated and stored at −20 °C until analysis [27].

Post-FST plasma corticosterone concentrations were determined using a competitive enzyme-linked immunosorbent assay (ELISA) kit (ENZO Life Sciences, catalog no. ADI-901-097, Lörrach, Germany). Sample extraction and analysis were performed according to the manufacturer’s protocol. Optical density was measured at 405 nm using a Tecan Infinite 200 PRO microplate reader (Tecan Group Ltd., 8708 Männedorf, Switzerland), and data analysis was conducted using Microsoft Excel (Microsoft Corporation, Redmond, WA, USA).

### 2.11. Statistical Analysis

Given the exploratory nature of the experiment, we performed effect size and power calculations (Supplementary Table S2) using G*Power Version 3.1.9.6 [28]. Before statistical comparisons, data were tested for normal distribution by the Gaussian and the Kolmogorov–Smirnov test. All the data shown in this manuscript were normally distributed except for the test of interaction, which was transformed (Y = log(Y)) to make the data normally distributed. Clicker training data between day 1 and day 2 were evaluated by the Mann–Whitney U test. For all other data, we used three-way ANOVA (main factors: training of offspring, training of mothers, and sex of the offspring) followed by Tukey’s Honestly Significant Difference (HSD). Tukey’s HSD was chosen as the post hoc procedure because it controls the family-wise error rate for all pairwise comparisons within a factor, providing a balance between Type I and Type II error under a fixed-effects model. Statistical analyses were performed with GraphPad Prism, Version 9.4.1, for Windows (GraphPad Software, San Diego, CA, USA). The F values indicated the variance ratio between and within groups. Degrees of freedom are shown as subscripted F values. The level of significance is provided in each figure. In all the figures, the values were expressed as means ± SD. Raw data are provided in Supplementary Table S1.

## 3. Results

### 3.1. Effect Size and Power Calculation

We calculated the effect size and power calculations for all the variables (Supplementary Table S2): Voluntary Interaction (Effect size 0.343; Power: 0.63), Body Weight Gain (Effect size 0.366; Power: 0.65), EPM Distance (Effect size 0.515; Power: 0.93), OFT Center Time (Effect size 0.531; Power: 0.54), OFT Distance (Effect size 0.512; Power: 0.93), CORT (Effect size 1.336; Power: >0.99), CORT (Effect size 0.677; Power: 0.98), NBT (Effect size 0.013; Power: 0.05), SPT (Effect size 0.128; Power: 0.14), FST (Effect size 0.146; Power: 0.14). Despite the low power observed in some variables, we conducted the three-way ANOVA test to identify potential factor effects and group differences.

### 3.2. Development and Validation of the Clicker Training Protocol

The evaluation of clicker training success is based on the frequency of training behavior from offspring across the first two days. If the frequency of the training behavior increases, the conditioning was successful (Figure 2). Both sexes exhibited a significant increase in training frequency. Trained females (*p* = 0.0049) and males (*p* = 0.0194) showed a higher training frequency at day two compared to day one.

### 3.3. Behavioral Effects of Training in F1 Offspring (Interaction, Weight Gain, EPM, OFT, NBT, SPT, FST)

The data of the voluntary interaction test was not normally distributed and therefore transformed (Kolmogorov–Smirnov test with Dallal-Wilkinson-Lillie). The training of the offspring led to a significantly longer interaction duration (three-way ANOVA, *p* = 0.0416, F_(1,38)_ = 4.445) with the experimenter (Figure 3).

**Figure 4:**
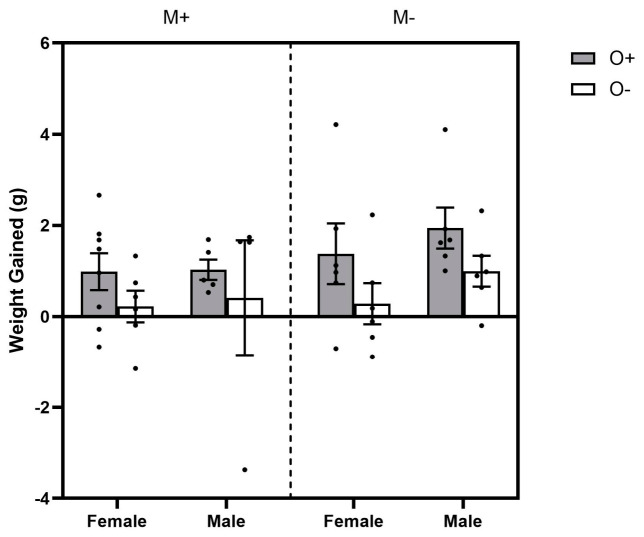
Weight gained by the offspring during the training period. It shows the weight gain of both sexes. We used three-way ANOVA for statistical analysis and multiple comparisons. M-O- group (*n* = 12), M+O- group (*n* = 10), M-O+ group (*n* = 12), M+O+ group (*n* = 13). Each point represents an individual mouse. Bars indicate the means ± SD.

The effect of clicker training on body weight was assessed by measuring weight changes before and after training (Figure 4). All mice gained weight during the training period. Training the offspring led to a significantly higher weight gain (three-way ANOVA, *p* = 0.0275, F_(1,39)_ = 5.248).

**Figure 5:**
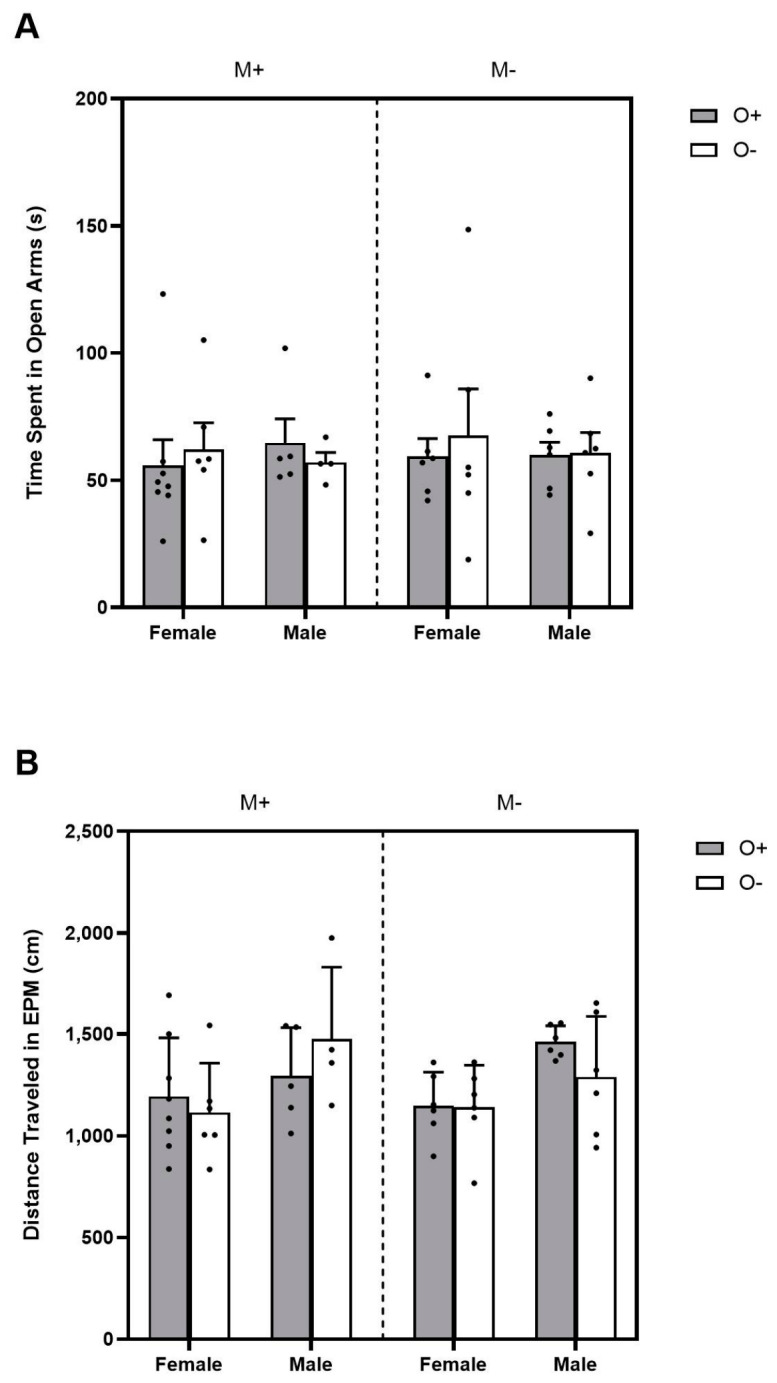
Time spent in open arms (**A**) and total distance traveled (**B**) in the EPM by the offspring. We used three-way ANOVA for statistical analysis and multiple comparisons. M−O− group (*n* = 12), M+O− group (*n* = 10), M−O+ group (*n* = 12), M+O+ group (*n* = 13). Each point represents an individual mouse. Bars indicate the means ± SD.

Neither the clicker training nor the sex of the offspring showed a significantly longer (three-way ANOVA) duration time in the open arms of the EPM in this exploratory study. Likewise, within the exploratory framework of this study, offspring training appeared to influence the total distance traveled in the EPM (Figure 5). In contrast, the sex of the offspring showed a significant effect on the distance traveled (three-way ANOVA, *p* = 0.0026, F_(1,39)_ = 10.34).

**Figure 6:**
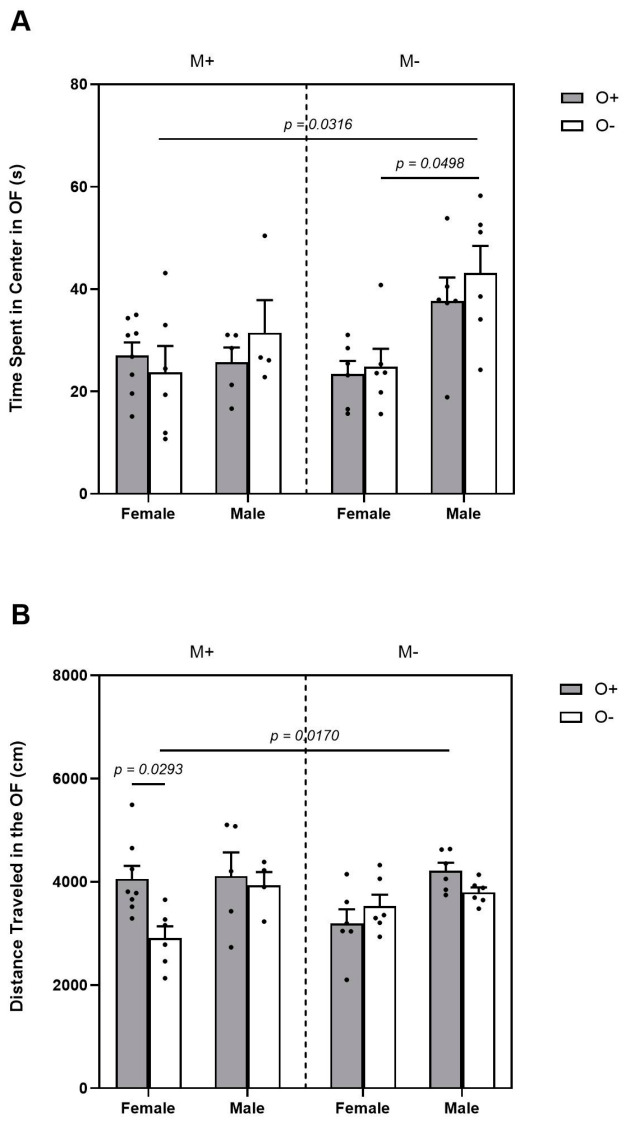
Time spent in the center of the OF test (**A**) and total distance traveled (**B**) by the offspring. We used three-way ANOVA for statistical analysis and multiple comparisons. (**A**) The sex of the offspring influences the time spent in the center, and the interaction of the sex of the offspring and the training of the mothers. (**B**) The sex of the offspring also influences the total distance traveled. The interaction of training mothers, training offspring, and their sex also influences the distance. M−O− group (*n* = 12), M+O− group (*n* = 10), M−O+ group (*n* = 12), and M+O+ group (*n* = 13). Each point represents an individual mouse. Bars indicate the means ± SD. The exact *p*-values are provided when significant differences are given.

The evaluation of the OF also showed no significant difference in the duration in the center (Figure 6) within this exploratory study. The training of the offspring was associated with differences in the duration of time spent in the center of the OF. However, sex had a significant effect on the length of stay in the center of the OF (three-way ANOVA, *p* = 0.0020, F_(1,39)_ = 10.97). The training of the offspring did not show an influence on the distance covered. However, when sex is considered, there is a significant difference in the distance covered (three-way ANOVA, *p* = 0.0027, F_(1,39)_ = 10.26). The interaction between sex, the training of the mothers, and the training of the offspring also indicated an effect on the distance traveled in the OF (three-way ANOVA, *p* = 0.0244, F_(1,39)_ = 5.486). These findings are interpreted as exploratory and hypothesis-generating.

The nest-building test showed no differences in the nest score of the training (three-way ANOVA, *p* = 0.9374, F_(1,39)_ = 0.0062) and the sex of the offspring (three-way ANOVA, *p* = 0.3281, F_(1,39)_ = 0.9809) within this exploratory study (Supplementary Table S1). The sucrose preference test showed no differences in the consumption of sucrose solution between the training (three-way ANOVA, *p* = 0.4237, F_(1,39)_ = 0.6537) and the sex (three-way ANOVA, *p* = 0.8066, F_(1,39)_ = 0.061) of the offspring (Supplementary Table S1).

The FST test showed no differences in immobility duration between the training (three-way ANOVA, *p* = 0.4285, F_(1,30)_ = 0.6443) and sex (three-way ANOVA, *p* = 0.2117, F_(1,30)_ = 1.628) of the offspring (Supplementary Table S1).

### 3.4. Physiological Stress Markers: Corticosterone

**Figure 7:**
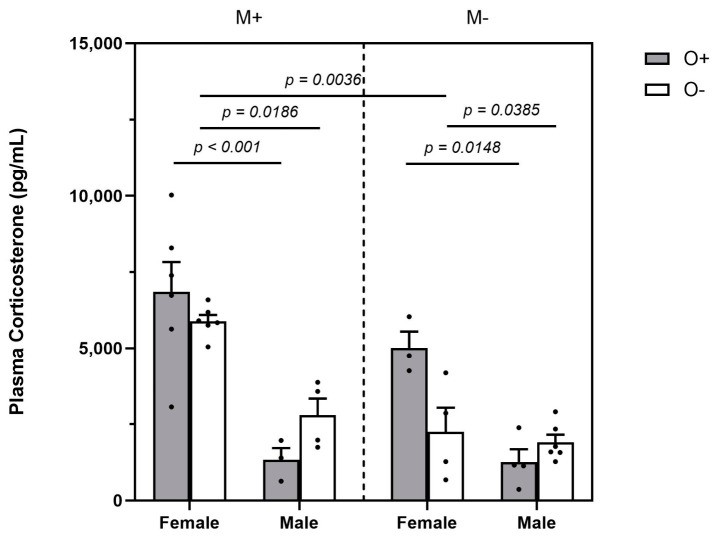
Corticosterone concentrations in the blood of the Crl:CD1(ICR) offspring. We used three-way ANOVA for statistical analysis and multiple comparisons. The training of the mothers and the sex of the offspring influence the plasma corticosterone. M−O− group (*n* = 10), M+O− group (*n* = 10), M−O+ group (*n* = 7), M+O+ group (*n* = 9). Each point represents an individual mouse. Bars indicate the mean ± SD. The exact *p*-values are provided when significant differences are given.

The sex of the offspring (three-way ANOVA, *p* < 0.0001, F_(1,28)_ = 50.05) has a significant influence on the post-FST plasma corticosterone (Figure 7). In addition, there is a significant difference in the factor sex and the training of the offspring (three-way ANOVA, *p* = 0.0030, F_(1,28)_ = 10.58).

### 3.5. Maternal Training Effects

Overall, the maternal effects (M+) are selective and predominantly exploratory in the data. In this pilot experiment, most behavioral parameters of the F1 offspring were not clearly influenced by the training of the dams. In the voluntary interaction test, maternal training did not influence interaction duration with the experimenter. The increased interaction time was primarily associated with direct offspring training (Figure 3).

Similarly, maternal training had no effect on body weight gain (Figure 4). Maternal training and sex of the offspring were not associated with marked differences in weight gain. Similarly, maternal training had no effect on nest building performance, sucrose preference, or immobility duration in the FST (Supplementary Table S1). In the Elevated Plus Maze (EPM), no main effect of maternal training was observed for either time spent in the open arms or total distance traveled. In the Open Field Test (OF), maternal training alone did not significantly influence time spent in the center or total distance traveled. Nevertheless, a potential interaction between offspring sex and maternal training was detected for time spent in the center (three-way ANOVA, *p* = 0.0314, F_(1,39)_ = 4.984). In addition, a potential interaction between offspring sex, maternal training, and offspring training was observed for distance traveled in the OF (three-way ANOVA, *p* = 0.0244, F_(1,39)_ = 5.486). These findings may indicate sex-dependent effects of maternal training on exploratory behavior and locomotor activity. The clearest potential maternal effect was detected in the physiological stress response. Maternal training influenced post-FST plasma corticosterone concentrations (Figure 7) in the offspring (three-way ANOVA, *p* = 0.0013, F_(1,28)_ = 12.82). Furthermore, interactions were observed between maternal training and offspring sex (three-way ANOVA, *p* = 0.0178, F_(1,28)_ = 6.337). As stated, given the exploratory pilot nature of the study, these results should be interpreted cautiously and considered hypothesis-generating.

## 4. Discussion

Based on the effect size and power calculations, rather than a definitive conclusion, this study provides preliminary evidence for potential transgenerational effects of clicker training in Crl:CD1(ICR) mice and highlights the complexity of the interactions between positive reinforcement training, sex-specific behavioral responses, and maternal transmission. Contrary to our initial hypothesis, the findings did not provide clear evidence that training of the dams reduced anxiety-like behavior or physiological stress markers in the offspring. Instead, the results reveal a complex and not straightforward pattern that complicates their interpretation in the context of animal welfare, while at the same time contributing valuable insights to the growing body of literature on refinement strategies in laboratory animal science. Several methodological limitations must, however, be considered when interpreting these findings. The discrepancy between the three-way ANOVA results and Tukey post hoc analyses for key outcome measures suggests limited statistical power to detect the relatively small effect sizes that are typical for this type of research (Supplementary Table S1). In addition, although the behavioral test battery was comprehensive, it primarily relied on paradigms designed to identify pathological states, potentially underestimating improvements in positive affective states or adaptive coping capacities. A critical limitation of the present study is the timing of blood collection for CORT analysis. Samples were obtained approximately 15 min after the FST, meaning that measured concentrations reflect acute stress reactivity in response to forced swimming rather than resting HPA-axis tone. This prevents any direct comparison between groups with respect to chronic stress burden or baseline welfare state and represents a fundamental constraint on the interpretation of the CORT data presented here. Future studies should include a separate baseline CORT measurement from a non-stressed cohort. Furthermore, because blood sampling followed directly after the behavioral testing battery, the procedures themselves may have confounded both physiological and behavioral responses, thereby masking more subtle group-specific differences. Taken together, these limitations emphasize the need for future studies employing larger sample sizes, more sensitive welfare-oriented endpoints, and longitudinal physiological assessments to better characterize the potential long-term and transgenerational effects of positive reinforcement training.

### 4.1. Context-Specific Benefits of Clicker Training (Offspring Training)

Training of the offspring increased the time of interaction between the mice and the experimenters. Successful clicker training seems to follow principles of operant conditioning in laboratory rodents [12]. Leidinger et al. (2017) [11] showed that positive reinforcement training improves laboratory conditions for animals by reducing stress and anxiety-like behaviors. Our observation of enhanced voluntary interaction with experimenters suggests that clicker training directly in the offspring produces targeted improvements in social engagement and exploration (e.g., interaction with the experimenter and OF test) rather than generalized behavioral changes. This context specificity is particularly noteworthy when contrasted with the absence of effects in classical anxiety paradigms (EPM, NB, SPF tests). The dissociation between improved human–animal interactions and unchanged performance in the mentioned tests is difficult to interpret with our current findings; however, recent evidence suggests that reward-based training activates specific neural circuits associated with teaching complex behaviors, which may not overlap substantially with other cognitive processes [29]. Given the complexities of potential interactions between maternal and offspring training as well as sex-specific effects, these assumptions warrant further mechanistic investigation in future studies. Moreover, the exclusive focus on offspring behavioral analyses, while consistent with the primary study objectives, limited insights into potential training effects in the maternal generation. As a consequence, direct comparisons of training efficacy across generations were not possible, thereby constraining the broader contextual interpretation of potential transgenerational outcomes.

### 4.2. Maternal Training

The finding that maternal training was associated with decreased center time during offspring open field exploration and increased post-FST corticosterone concentrations challenged our initial assumptions regarding potential transgenerational welfare benefits. However, these findings must be interpreted with caution in light of the study limitations. In particular, the experimental subjects originated from only two dams, increasing the likelihood of littermate effects and limiting the genetic and environmental diversity represented. While efforts were made to minimize husbandry and experimental effects, replication across additional litters is necessary to confirm the robustness of the observed effects and enhance generalizability. Accordingly, the findings should be interpreted as preliminary exploratory data rather than definitive evidence.

The overall limited transmission of potential training effects from mothers to offspring further suggests that such effects may be highly context-dependent and influenced by factors including timing, environmental conditions, and the specific nature of the parental experience. Within the scope of the present study, maternal training alone did not result in clear improvements in offspring welfare. Nevertheless, the data indicated possible interaction effects between sex, maternal experience, and offspring training. In particular, the combination of maternal training and training of female offspring was associated with increased post-FST corticosterone concentrations. Importantly, these values remained below the reference ranges reported in the literature and therefore did not indicate impaired welfare [30,31].

Overall, the findings suggested that training of the offspring themselves exerted a stronger influence on the investigated parameters than potential maternally transmitted effects. It should also be considered that, under the present non-stressful housing conditions, only minor training effects were expected, which is consistent with previous studies reporting similarly modest effects in low-stress environments. In addition, the relatively short training duration of only four days may have been insufficient to induce stable behavioral or physiological adaptations.

Further methodological limitations restricted a more detailed mechanistic interpretation of the results. Additional stress-related parameters, including adrenocorticotropic hormone (ACTH), corticosteroid-binding globulin, and glucocorticoid receptor expression, were not assessed, thereby preventing a comprehensive evaluation of hypothalamic–pituitary–adrenal (HPA) axis function. Moreover, limitations in study duration and available resources prevented longitudinal assessments, dose–response investigations of different training protocols, and evaluation of varying temporal intervals between maternal training and breeding. In particular, the eleven-week interval between maternal training and offspring exposure may have been sufficient to attenuate potential epigenetic or hormonal influences. Taken together, these findings highlight the complexity of potential transgenerational training effects and emphasize the need for future studies incorporating larger sample sizes and more comprehensive physiological analyses to further elucidate the underlying mechanisms.

### 4.3. Sex Differences in Training

Sex-specific differences manifested both behaviorally [32,33] and physiologically (corticosterone [34,35]), highlighting the importance of considering sex as a critical variable in research. The differential effects of clicker training on sex behavior have been reported previously; however, that study assessed the effects of clicker training directly in mice without parental interventions [23].

Female offspring tended to show higher distance traveled in the OF test and higher post-FST corticosterone concentrations, particularly when their mothers had received training. The sex-specific nature of this response reflects well-established differences in hypothalamic–pituitary–adrenal axis function between males and females. Female rodents consistently demonstrate greater post-FST corticosterone responsivity to both positive and negative stimuli, suggesting heightened physiological sensitivity rather than gender-specific vulnerability to stress. This elevated responsiveness may represent enhanced physiological flexibility and adaptability in females from trained mothers, although this interpretation remains speculative and requires further investigation.

Male offspring displayed distinct patterns, with their own training producing different effects compared to those associated with maternal training. These sex-specific patterns likely reflect evolutionary differences in how males and females respond to environmental challenges and opportunities. Understanding these differences is crucial for developing optimized training protocols that account for biological sex variations. Understanding these differences is crucial for developing optimized training protocols that account for biological sex variations.

## 5. Future Perspectives

Studying the effects of clicker training on transgenerational effects offers new research opportunities for understanding animal welfare and refinement research. Our findings suggested that training of the offspring themselves played a decisive role in improving physiological parameters (as previously demonstrated in other studies), whereas maternal effects could not be clearly interpreted under the present experimental conditions.

We consider that future research should focus on developing training protocols in different stages of reproductive periods, including pregnancy, focusing on implementing continuous stress monitoring during training, and assessing mechanistic pathways associated with the effects of clicker training on maternal traits transmitted to F1-generation. In addition, we recommend integrating physiological stress markers, beyond corticosterone levels, with behavioral assessments, providing actual feedback on intervention effects, enabling a more robust interpretation to optimize welfare outcomes.

Future studies should investigate whether applying clicker training under more challenging or stressful conditions could influence offspring welfare. However, the expected effects are likely to be small and therefore difficult to detect, as also reflected in the inconsistent findings reported in studies on handling procedures and related interventions. Consequently, extensive research efforts and larger datasets will be required to reliably identify such effects.

In addition, while CD1 mice represent a relatively robust and genetically variable model, future studies should include other mouse strains to account for strain-specific stress responsiveness. For example, more stress-sensitive strains such as BALB/c mice may respond differently to training procedures, as stress-related phenotypes are often strongly strain-dependent [36]. Expanding research across multiple strains will therefore be essential to obtain a comprehensive understanding of training effects.

Finally, investigating the epigenetic mechanisms underlying potential transgenerational effects will be useful to identify key points of intervention and to optimize training protocols that maximize welfare benefits across generations. Overall, substantially more empirical data will be required before firm conclusions about the transgenerational welfare effects of clicker training can be drawn.

## 6. Conclusions

The present pilot study provided exploratory insights into the effects of clicker training on CD1 laboratory mice and its potential transgenerational impact. Training of the offspring themselves significantly influenced welfare markers parameters, whereas maternal training alone did not produce clear or consistently interpretable benefits under the present experimental conditions, at least in this experiment. Findings also revealed sex-specific responses, highlighting the importance of considering sex in the design of welfare interventions. However, given the limited sample size (two dams per group), these results should be interpreted as preliminary exploratory observations and not as statistically robust inferences.

Importantly, we were able to establish a standardized clicker training protocol that can be reliably applied to the CD1 mouse strain, providing a framework material for future refinement protocols. Overall, as previously reported, clicker training appeared to offer targeted improvements in social engagement and exploration without inducing generalized stress; however, the limited transmission of maternal effects needs to be carefully timed and applied under context-specific interventions, with adequate systematic tracking. The present data provide preliminary, exploratory evidence that requires further analysis. Future research should aim to optimize training protocols, investigate underlying epigenetic mechanisms, and integrate more physiological markers of stress monitoring and their relationship with behavioral assessments to maximize welfare benefits provided by clicker training.

## Figures and Tables

**Table 1:** Timeline for clicker training.

Day 1	Day 2	Day 3	Day 4	Day 5	Day 6
Introduction of the reward		Introducing the click & 1st training	2nd training	3rd training	4th training

## Data Availability

Data is available as Supplementary Tables S1 and S2.

## Supplementary Materials

The following supporting information can be downloaded at: https://www.mdpi.com/article/10.3390/ani16111642/s1, Table S1: Raw Data; Table S2: Effect Size and Power Calculations.

## Abbreviations

The following abbreviations are used in this manuscript:

OF	Open Field
EPM	Elevated Plus Maze
NBT	Nest Building Test
SPT	Sucrose Preference Test
CORT	Corticosterone
FST	Forced Swim Test
BW	Bodyweight
M+O+	Trained offspring from trained mothers
M−O+	Trained offspring from untrained mothers
M+O−	Untrained offspring from trained mothers
M−O−	Untrained offspring from untrained mothers
